# Nitrate removal from aqueous solutions by γ-Al_2_O_3_ ultrafiltration membranes

**DOI:** 10.1016/j.heliyon.2017.e00498

**Published:** 2018-01-18

**Authors:** M. Breida, S. Alami Younssi, A. Bouazizi, B. Achiou, M. Ouammou, M. El Rhazi

**Affiliations:** Laboratory of Materials, Membranes and Environment, Department of Chemistry, Faculty of Sciences and Technologies of Mohammedia, University Hassan II of Casablanca, BP 146, Mohammedia 20650, Morocco

**Keywords:** Environmental science, Chemical engineering, Materials science, Chemistry

## Abstract

In the framework of understanding the transport mechanism that governs the filtration of NO_3_^−^ solution through a *γ*-Al_2_O_3_ membrane with a nominal pore size of 5 nm at low ultrafiltration, a series of various types of nitrate solutions and operating conditions were investigated. The effect of filtration parameters such as pH, applied pressure and NO_3_^−^ concentration on the selectivity and permeability of the membrane were studied using binary solutions (KNO_3_, NaNO_3_, Ca(NO_3_)_2_ and Mg(NO_3_)_2_) and ternary solutions ((NaNO_3_ + KNO_3_), (NaNO_3_ + Ca(NO_3_)_2_) and (Mg(NO_3_)_2_ + Ca(NO_3_)_2_). The experimental filtration results showed that high NO_3_^−^ rejection was observed when pH was close to the point of zero charge of the membrane for both binary and ternary solutions. NO_3_^−^ rejection increased with an increase of applied pressure. The rejection gradually decreased when the initial NO_3_^−^ concentration increased. It appeared that the valency and hydrated radius of associated cation had a dramatic effect on NO_3_^−^ rejection, with the divalent cations being more rejected than monovalent cations. In order to get to natural water complexity, three different samples of mineral water doped with NO_3_^−^ from two different sources were studied at optimized operating conditions (25 ppm of NO_3_^−^ and 6 bar). Experimental results demonstrated that NO_3_^−^ rejection strongly depended upon the total mineralization and the presence of divalent anions in solution. In addition, the obtained results showed the potential use of *γ*-Al_2_O_3_ ultrafiltration membrane for denitrificatoin of contaminated water especially in Moroccan agricultural areas.

## 1 Introduction

Industrialization and urbanization are two transformational forces that have shaped the past century, and which are still contributing to environmental pollution, making the access to water of adequate quality for human consumption a relevant problem and an environmental priority. Water pollution can have a direct impact on the social organization and is currently the object of binding norms that have become increasingly severe.

In this context, great attention was paid recently to nitrate (NO_3_^−^) as it is one of the main hazards in groundwater, and most aquifers in agricultural areas are affected by this contaminant [1, 2]. More recently, reports have shown that increased levels of NO_3_^−^ in groundwater are associated with many adverse health effects, the most important effects are the stomach cancer diseases by its capacity in stimulating the carcinogenic nitrosamines formation [3, 4], and methemoglobinemia commonly known as blue baby syndrome [5, 6]. The presence of NO_3_^−^ has also been correlated to an increase of plasma testosterone concentrations in female alligators and others [7]. Moreover, the accretion of NO_3_^−^ in receiving medium causes many ecological and environmental problems inducing the eutrophication and seasonal hypoxia [8, 9, 10], thus destroying natural habitats and altering river ecology. In order to overcome these issues, limits were established by different organizations. The European Union (EU) and the European Environment Agency (EEA) have set a limit of 50 mg/L [11]. The EPA (U.S. Environmental Protection Agency), on the other hand, set the maximum contaminant level for NO_3_^−^ in drinking water to 45 mg/L, or 10 mg/L as NO_3_-N. The World Health Organization (WHO) has set a higher limit of 50 mg/L [12], which is identical to the Moroccan standard [13], for surveillance and monitoring the water in public supply networks.

As a result, a number of methods were used for the removal of NO_3_^−^ contamination. The top three treatment methods which were applied full-scale for NO_3_^−^ removal involves ion exchange, electrodialysis and reverse osmosis [14, 15]. Each process appears to have a number of disadvantages and limitations. The use of one method over another may depend on several factors such as the cost of processing, ease of reproducing, the added value along with the mode of use of the obtained treated water and the occurrence, or lack thereof, of harmful side products. An intensive research on cost effective and efficient NO_3_^−^ removal techniques has been the focus of many recent studies especially on the use of membrane techniques as better alternatives to traditional treatment systems since membranes offers a high efficiency in the removal of pollutants that meets high environmental standards [16]. With regards to membrane material, ceramic membranes have a number of advantages such as a relatively narrow pore size distribution and a higher porosity (resulting in better separation characteristics and a higher flux), a higher mechanical stability, as well as higher durability and efficiency. Ceramic membranes are chemically more stable in harsh environments and can tolerate higher temperatures than polymeric membrane [17, 18]. The majority of scientific researchers are targeting the application of ultrafiltration (UF) membranes in pollution prevention [19]. McBain et al. [20], were the first to notice the retention of ionic species under certain conditions by UF membrane. The rejection of ionic species by charged tight UF is achievable due to the importance of electrostatic interactions (repulsion/attraction) between ions and the charged membrane surface [21]. Rejection by electrostatic interactions becomes possible when the concentrations of salts solutions are relatively low and the membranes are superficially charged [22, 23].

Taking these proprieties into consideration and also the various advantages of UF (higher flux and lower cost) over nanofiltration and inverse osmosis which are usually used for nitrate removal, were the driving reasons to research the nitrate retention possibility by a tight *γ*-Al_2_O_3_ ultrafiltration (*γ*-Al_2_O_3_ UF) membrane. The use of γ-Al_2_O_3_ UF membrane could be a potential solution that enables the effective removal of nitrates from polluted water, especially in Morocco, a country which is facing problem of water supply due to an increase in demand and a decrease of conventional resources as well as the lack of suitable treatment techniques. The occurrence of increased nitrate content within water intakes is a considerable problem in Moroccan agricultural areas due to the excess use of nitrate fertilizers (e.g. Souss-Massa basin, Essaouira Basin). The main objective of this study is to examine the possibility of high NO_3_^−^ rejection and thus providing an identification and understanding of rejection mechanisms. In order to ensure this, experiments were performed on various synthetic solutions of NO_3_^−^ of increasing complexity. This allowed the characterization of the influence of the associated cation and the comparison of the results obtained for the different solutions, while studying the variation of multiple factors (pH, pressure and the initial NO_3_^−^ concentration).

## 2 Experimental

### 2.1 UF membrane

The *γ*-Al_2_O_3_ UF membrane was used to remove nitrate from aqueous solutions. The tubular ceramic membrane was manufactured by Pall Corporation and its characteristics are reported in Table 1.

**Table 1:** The characteristics of the used *γ*-Al_2_O_3_ UF membrane.

Parameters	Values
Length	150 mm
Inner diameter	7 mm
Outer diameter	10 mm
Pore diameter	5 nm
Water permeability	5 L/m^2^ h bar
PZC	8–9 [24, 25]
Surface Charge	Amphoteric behavior
Volume flow rate	3.5714**^.^**10^−5^ m^3^/s

**Fig. 1:**
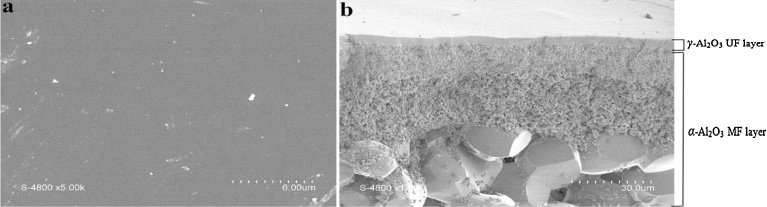
SEM of *γ*-Al_2_O_3_ membrane: top surface (a) and cross-section (b).

The morphological examination was carried out using a scanning electron microscope to verify the homogeneity of the filtering layer (Fig. 1). The *γ*-Al_2_O_3_ UF layer is deposited on the inner surface of tubular α-Al_2_O_3_ microfiltration (α-Al_2_O_3_ MF) support, exhibiting a good uniformity and adhesiveness.

### 2.2 Chemicals

NO_3_^−^ solutions were prepared in the laboratory with several electrolytes. The initial concentration was prepared by dissolving a known amount of salt in fresh pure water (ultrapure water type I with resistivity of 18.2 MΩ-cm was produced by Purelab Ultra, ELGA). All soluble salts, potassium nitrate (KNO_3_), sodium nitrate (NaNO_3_), calcium nitrate (Ca(NO_3_)_2_) and magnesium nitrate (Mg(NO_3_)_2_), along with reagents used in the experiments, were of analytical grade and acquired from Sigma Aldrich.

### 2.3 UF setup

**Fig. 2:**
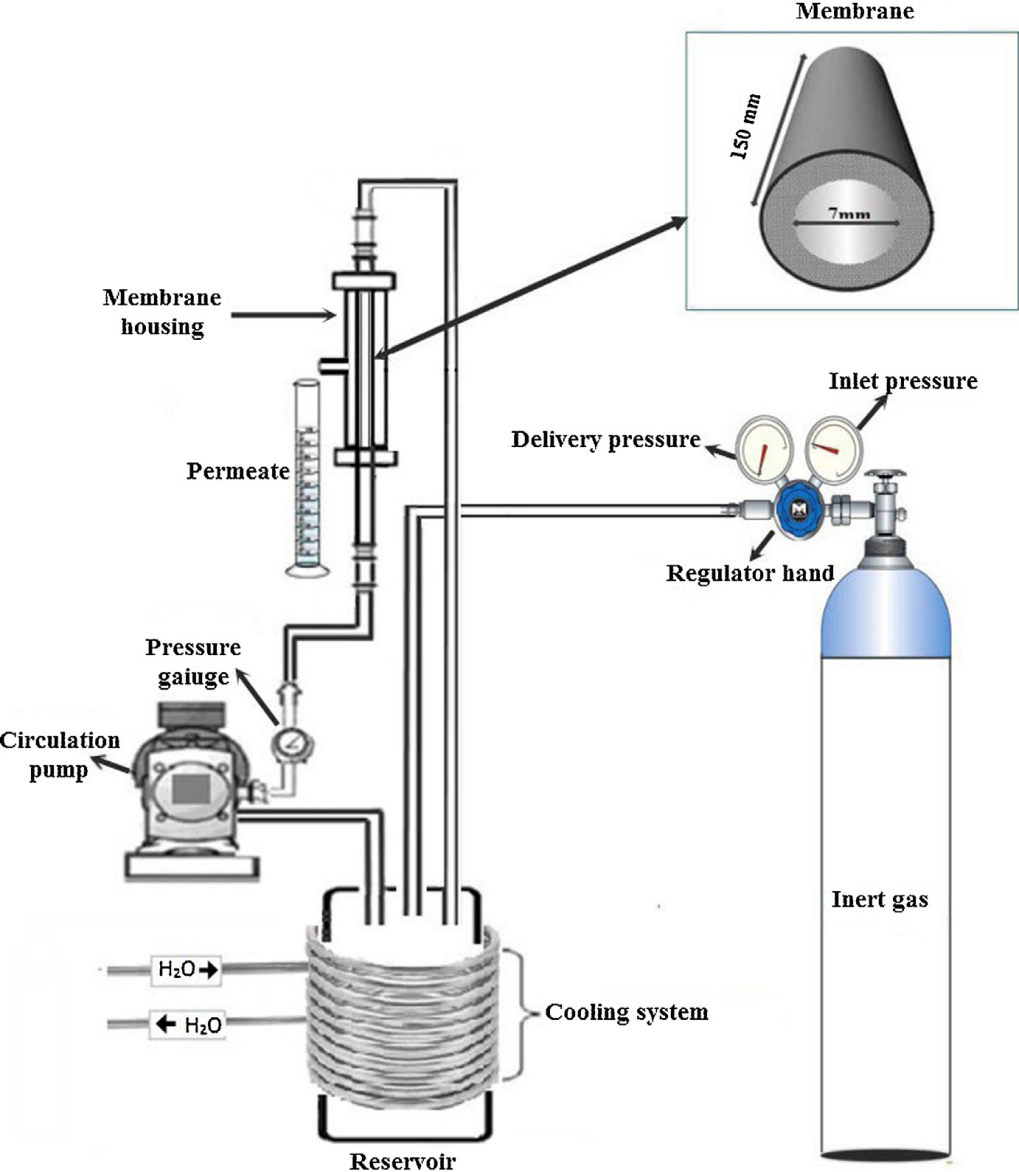
Schematic illustration of UF Pilot.

The membrane performance was determined using tangential filtration tests. All the filtration experiments were performed with a laboratory scale filtration pilot made from stainless steel (Fig. 2), comprised of feed tank of 3 L equipped with cooling system to maintain the feed solution temperature at constant value of 20 °C, a circulation pump, and membrane module. The applied pressure (ΔP) was adjusted by mean of compressed gas cylinder (Helium) using a needle-point valve. The applied pressure varied from 2 to 6 bar.

The permeate samples were collected and analyzed every 15 min during filtration time of 90 min. The reproductively of the membrane separation was evaluated by three filtration tests for each experiment and the result values giving in this work are the arithmetic mean. After each experiment, the membrane was cleaned with ultrapure water during 1 h in order to eliminate the impurities and to maintain the initial membrane performance.

The membrane performance was characterized by two important parameters that are the permeate flux Jp (L/h m^2^) and rejection rate R (%) which are respectively defined by Eqs. (1) and (2):

(1) $$J_{p}=V/A.t$$

(2) $$R=\left(1-C_{f}/C_{i}\right)\times 100$$

Where V (L) is the volume of permeate collected during a time interval t (h) and A (m^2^) is the effective membrane area. C_f_ and C_i_ (mg/L) are respectively the permeate and feed concentration of solute.

### 2.4 Analytical methods

The concentration of NO_3_^−^ was colorimetrically analyzed before and after filtration, by means of UV–vis spectrophotometer (UNICAM UV2 UV/Vis Spectrometer, ATi) at wavelength of 415 nm, according to the International Organization for Standardization (ISO 7890­3). The limit of detection of the method is 0.013 mg/L. Analyses of potassium (K^+^) and Sodium (Na^+^) were carried out using a Flame Photometer-Model PFP7 with limit of detection of 0.2 mg/L. The concentrations of magnesium (Mg^2+^) and calcium (Ca^2+^) were analyzed by complexometric titration-EDTA following French standard (Afnor–NF/T90-016 and NF/T90-003), with limit of detection of 0.05 mmol/L. The pH of solution was measured by pH meter-Seven compact (Mettler-Toledo GmbH, Analytical), with accuracy of ±0.05.

### 2.5 Operating procedure

The separation capability of the *γ*-Al_2_O_3_ membrane was studied by performing filtration experiments of binary and ternary solutions. It should be noted that binary solution corresponds to 1 cation/1 anion and ternary solution corresponds to 2 cations/1 anion. The study was divided in three parts.

Firstly, the effect of applied pressure and NO_3_^−^ feed concentration was investigated for monovalent cations (NaNO_3_ and KNO_3_) and divalent cations (Ca(NO_3_)_2_ and Mg(NO_3_)_2_) at a natural pH (Varied from 5.70 to 6.50). The applied pressure was varied from 2 to 6 bar and the feed concentration was in the range of 25–100 mg/L of NO_3_^−^. The influence of pH feed on membrane rejection was also studied in the pH range of 3–9 for feed concentration of 50 mg (NO_3_^−^)/L at pressure of 6 bar. The pH was adjusted by addition of HCl or NaOH solutions with a concentration of 1 M.

Secondly, the filtration of ternary NO_3_^−^ solutions containing monovalent cations (NaNO_3_ and KNO_3_) and divalent cations (Ca(NO_3_)_2_ and Mg(NO_3_)_2_) as well as monovalent and divalent cations (NaNO_3_ and Ca(NO_3_)_2_) were studied at different applied pressure at natural pH (5.70–6.50). The total concentration of NO_3_^−^ ions in the prepared solutions was studied over the range of 25–100 mg(NO_3_^−^)/L according to the NO_3_^−^ proportion shown in Table 2.

**Table 2:** Proportion of NO_3_^−^ in ternary feed solutions.

	Proportion (%) from Salt 1	Proportion (%) from Salt 2
[NO_3_^−^] in the feed (Salt 1 + Salt 2)	0	100
	25	75
	50	50
	75	25
	100	0

The filtration experiment was carried out at applied pressure of 2, 4 and 6 bar at natural pH and concentration of 50 mg(NO_3_^−^)/L. The effect of pH on NO_3_^−^ rejection was studied between pH 3 and pH 9 for the different ternary solutions (with a total concentration of 50 mg(NO_3_^−^)/L and a pressure of 6 bar).

The third step consisted of the study of three commercial mineral water at pressure of 6 bar and natural pH (pH 7.00). The mineral compositions of these water samples are shown in Table 3. Before filtration, an amount of nitrate salt equivalent to 25 mg(NO_3_^−^)/L was added to water samples using two NO_3_^−^ sources (NaNO_3_ and Ca(NO_3_)_2_).

**Table 3:** Mineral composition (mg/L) of commercial bottled water.

Concentration on mg/L	Sidi Ali water	Aïn Saiss water	Aïn Ifran water
Sodium	25.50	08.00	03.00
Calcium	12.02	63.50	67.73
Magnesium	08.70	35.50	40.61
potassium	02.80	01.00	01.00
Bicarbonates	103.70	372.00	402.60
Sulfates	41.70	03.80	05.13
Chlorides	14.20	19.80	10.65
Nitrates	0.10	07.00	05.18

Both for ternary and commercial water samples, the flux was measured every 15 min, and analyzed afterwards. The characteristics of the different ions used in this study are presented in Table 4.

**Table 4:** Properties of related cations and anions.

Ion	Ionic weight (Da)	Ionic radius (nm)	Hydrated radius (nm)	Hydrated energy (Kg/mol)	Diffusivity (10^−9^ m^2^/s)
Na^+^	23.0	0.117 [26]	0.358 [26]	−405 [27]	1.334 [28]
Mg^2+^	24.3	0.072 [26]	0.428 [26]	−1922 [27]	0.706 [28]
Ca^2+^	40.0	0.100 [26]	0.412 [26]	−1592 [27]	0.792 [28]
Cl^−^	35.5	0.194 [26]	0.332 [26]	−363 [27]	2.032 [28]
SO_4_^2−^	96.0	0.290 [27]	0.379 [27]	−1145 [27]	1.065 [28]
K^+^	39.0	0.149 [26]	0.331 [26]	−321 [27]	1.957 [28]
NO_3_^−^	63.0	0.189 [29]	0.340 [26]	−328 [27]	1.902 [28]

## 3 Results and discussion

### 3.1 Influence of solution pH

#### 3.1.1 Influence of solution pH on NO_3_^−^ in binary solutions

The surface charge of membrane generally depends on the pH of the feed solution. The study of this key factor allowed recognition of the efficiency of membrane separation in the process of ionic species removal [21, 30, 31].

The hydrated surface of alumina is known by amphoteric behavior. This property allows controlling the sign and the charge density of membrane surface by pH control [32]. The point of zero charge (pzc) matches the pH when the surface charge is null (pH_pzc_). On other words, pzc is the value for which the electric charges of the fixed cations globally neutralize anions. At a pH > pzc, the acidic dissociation of the surface hydroxyl groups leads to a negative surface charge attributed to the presence of AlO^−^ groups. Whereas, the positive charge when the pH < pzc is interpreted based on proton addition to the neutral aquocomplex due to the existence of AlOH_2_^+^ groups [33, 34] according to the following reactions (Eqs. (3) and (4)):

(3) Al—OH + H_2_O ↔ Al—OH^+^_2_ + OH^−^

(4) Al—OH + H_2_O ↔ Al—O^−^+ H_3_O^+^

**Fig. 3:**
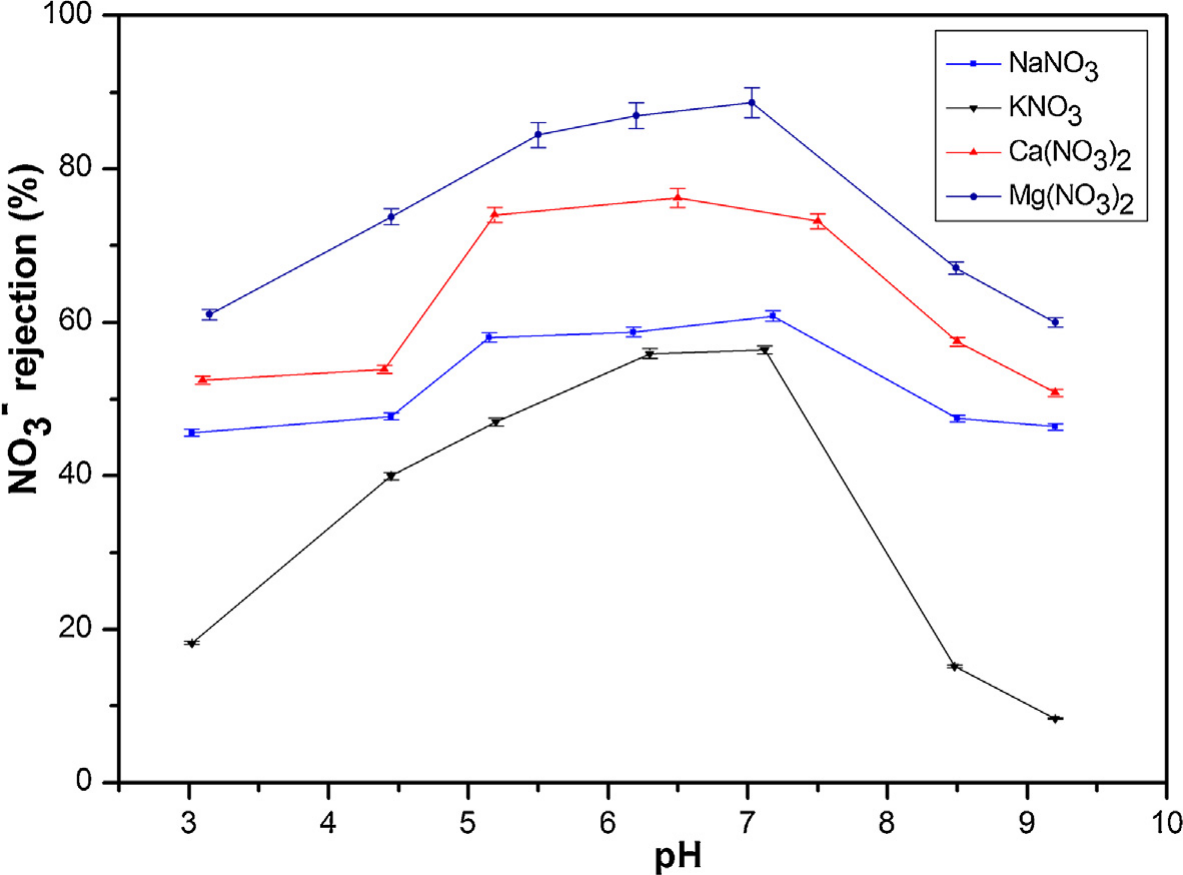
Influence of feed pH on NO_3_^−^ rejection (%) in binary solutions KNO_3_, NaNO_3_, Ca(NO_3_)_2_, Mg (NO_3_)_2_ at ΔP = 6 bar and C_i_ = 50 mg(NO_3_^−^)/L.

Fig. 3 presents the effect of feed solution pH on NO_3_^−^ rejection at applied pressure of 6 bar and initial concentration of 50 mg(NO_3_^−^)/L. In acidic pH range, the NO_3_^−^ rejection increased until reaching a maximum around a pH between 6.5 and 7.5. However, the rejection decreased with the pH in alkaline range.

Strong interactions developed between the divalent cations and the positively charged membrane (pH < pzc) which resulted in high cation rejection. The NO_3_^−^ rejection also increased when pH increase due to electroneutrality consideration, illustrated by a rejection rate which exceeded 80% for divalent cation Mg(NO_3_)_2_, and 50% for monovalent cation NaNO_3_. The ion separation is highly governed by the Donnan exclusion (charge effect) [31, 35]. Eyraud et al. explained this behavior by the distribution variation on the membrane surface charge as function of pH [36]. At pH_pzc_, the membrane is uncharged (no electrostatic repulsion), the selectivity is governed by sieving mechanism based on ion size. For pH > pzc, as shown in Fig. 3 a decrease in nitrate rejection occurred at pH 9.2, this decrease in salt retention observed when the pH increased can be explained by a decrease in the positive charge of *γ*-Al_2_O_3_ membrane in the presence of the different electrolytes [22]; which facilitates the passage of cations through the membrane.

#### 3.1.2 Influence of solution pH on NO_3_^−^ in ternary solutions

**Fig. 4:**
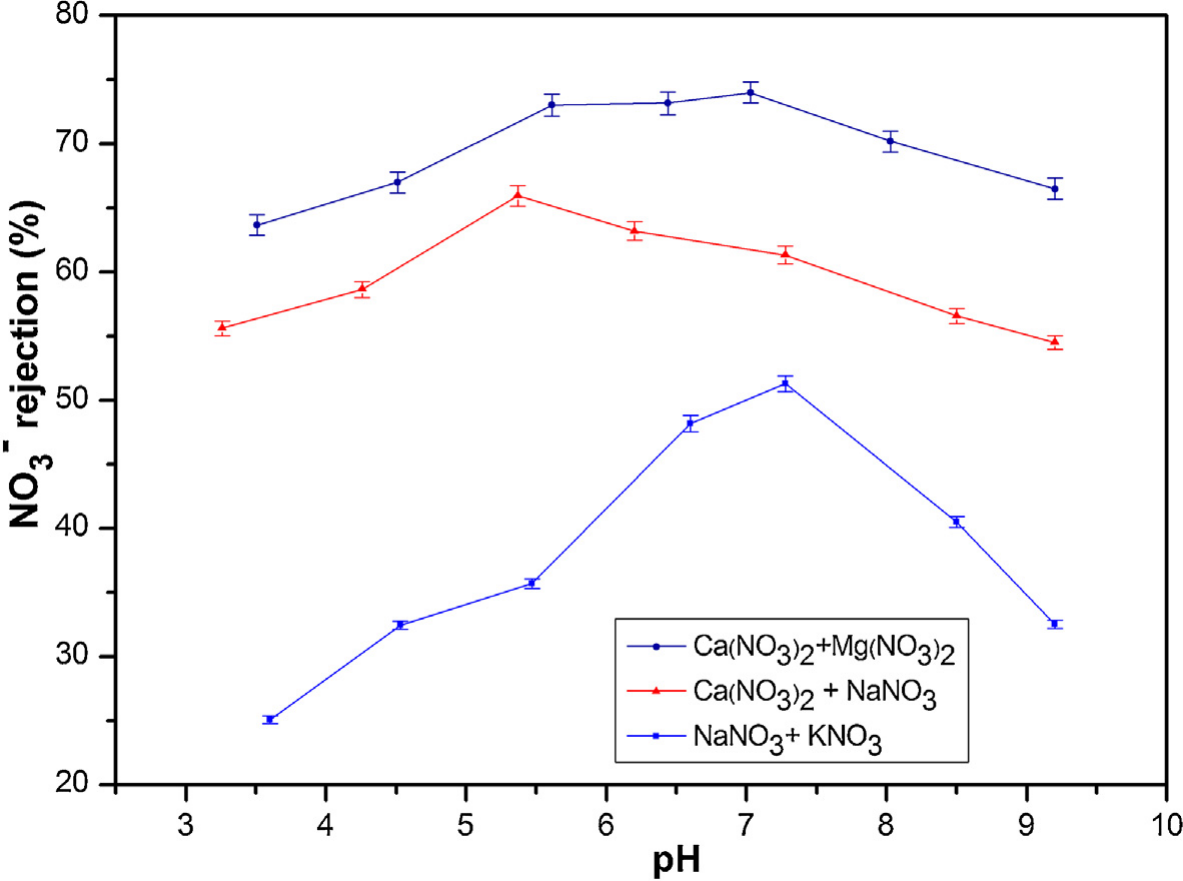
Influence of feed pH on NO_3_^−^ rejection (%) in ternary solutions (NaNO_3_ + KNO_3_) (NaNO_3_ + Ca(NO_3_)_2_), (Mg(NO_3_)_2_ + Ca(NO_3_)_2_) (at fixed ΔP and C_i_ of 6 bar and 50 mg (NO_3_^−^)/L).

In the case of ternary solutions, the rejection rate strongly depended upon pH and the type of ions present in the solution. Fig. 4 displays the influence of feed pH on NO_3_^−^ rejection in ternary solutions at applied pressure of 6 bar and initial concentration of 50 mg(NO_3_^−^)/L. The results shows maximum rejection at pH close to the pH_pzc_, for the different ions, in the case of mixtures of two monovalent cations (NaNO_3_ + KNO_3_) and two divalent cations (Mg(NO_3_)_2_ + Ca(NO_3_)_2_) (demonstrated by NO_3_^−^ rejection equal to 51% and 74% respectively). However, for monovalent and divalent salt (NaNO_3_ + Ca(NO_3_)_2_) the best rejection is at pH 5.37. This result is related to capacity of Ca^2+^ to form surface complexes with the *γ*-Al_2_O_3_ surface groups (AlOH_2_^+^ or AlO^−^) and simultaneously shifting pH toward high or low pH values [37]. As in the case of binary solutions, the results obtained in ternary solutions were significantly affected by pH. Authors explained this finding by the fact that the membrane charge density and consequently the exclusion by Donnan varies as a function of the pH.

**Fig. 5:**
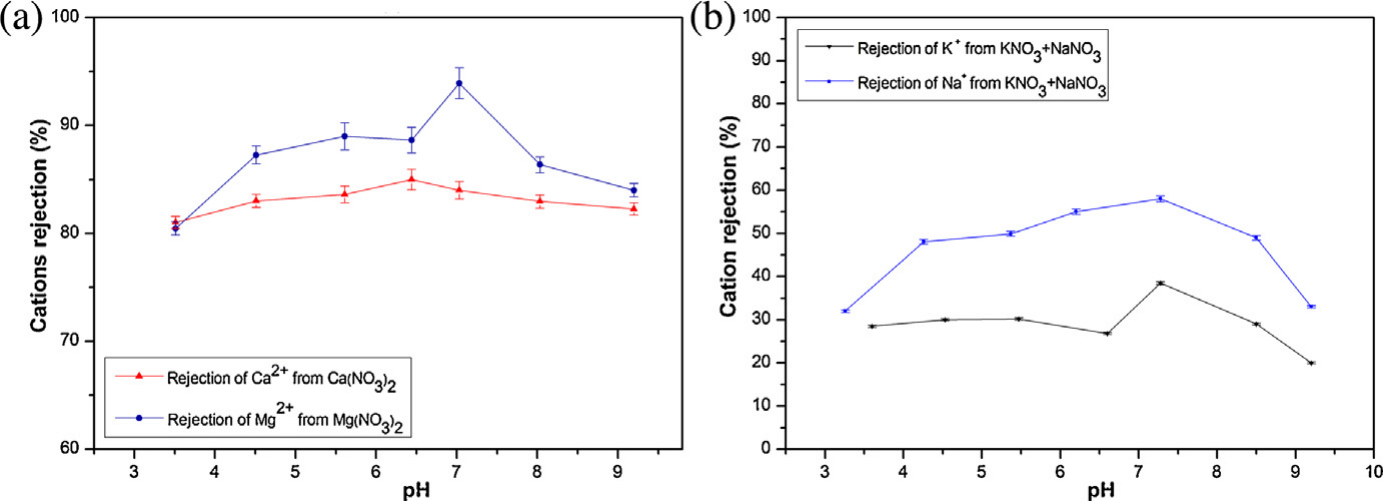
Influence of feed pH on cation rejections (%) in binary solutions Ca(NO_3_)_2_, Mg(NO_3_)_2_ solutions (a) and ternary solutions (NaNO_3_ + KNO_3_) (b) (at fixed ΔP = 6 bar and C_i_ = 50 mg(NO_3_^−^)/L).

Fig. 5 illustrates the cation rejections (Ca^2+^ and Mg^2+^ rejections in binary solutions (Fig. 5a), and Na^+^, K^+^ in ternary solutions (Fig. 5b)) as function of feed solution pH. The rejection of cations follows the same behavior of nitrate rejection (Fig. 5) that manifests by high rejection at pH close to the pH_pzc_ (between pH 6.5 and pH 7.5) (with rejection equal to 83% and 93% respectively for Ca^2+^ and Mg^2+^ and 58% and 38% respectively for Na^+^ and K^+^).

When the membrane is soaked in aqueous solution filled with electrolyte at a defined pH value, it can obtain a surface charge meanly due to a simultaneous acid/base dissociation of hydrophilic functional groups. The presence of charge groups (positive/negative) on the membrane surface causes an increase in the electrostatic interaction phenomenon between the dissolved ions and surface, known as counter-ion site binding [38, 39].

#### 3.1.3 Influence of feed pH on permeates flux

**Fig. 6:**
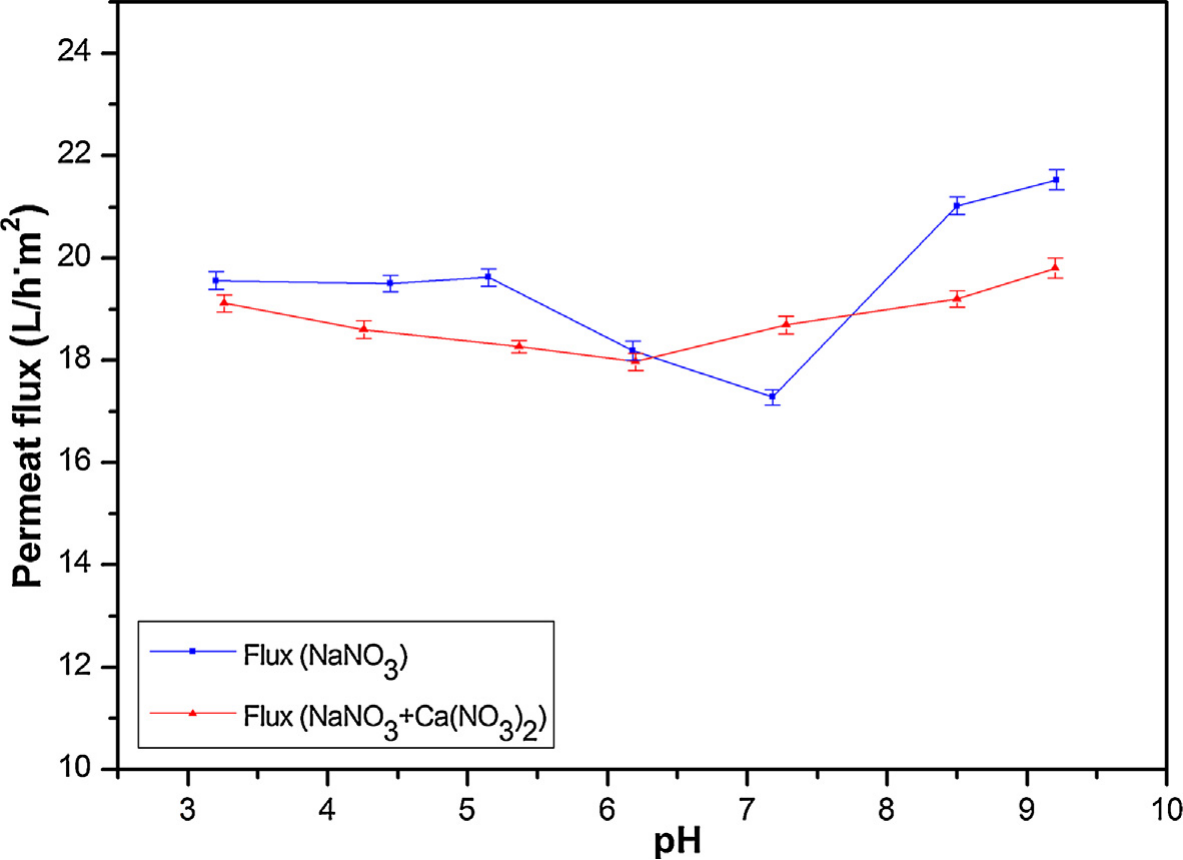
Permeate fluxes versus pH (at concentration of 50 mg(NO_3_^−^)/L and pressure of 6 bar).

Fig. 6 shows the permeate flux of the *γ*-Al_2_O_3_ membrane versus pH, ranging between pH 3 and pH 9 and for fixed concentration of 50 mg(NO_3_^−^)/L and pressure of 6 bar. No marked effect was observed in the variation of flux as a function of pH. The flux slightly decreased with increasing pH until a value of 7.2 which was near the pzc of the studied membrane and matched a high NO_3_^−^ rejection. When the membrane surface possessed a positive charge, the electrostatic repulsion between the membrane charge and cations increased with increasing pH, which resulted in a slight decrease of fluxes. However, when pH is higher than pH_pzc_ the permeate flux increased with pH. A similar finding has been reported with α-Al_2_O_3_ and *γ*-Al_2_O_3_ membranes attributing the increase of flux with the pH increase to three considerations (electrostatic interaction, pore size and osmotic pressure gradient) happening at the surface of the membrane [40, 41, 42, 43].

### 3.2 Effect of applied pressure

#### 3.2.1 Pressure effect on permeate flux and NO_3_^−^ rejection

**Fig. 7:**
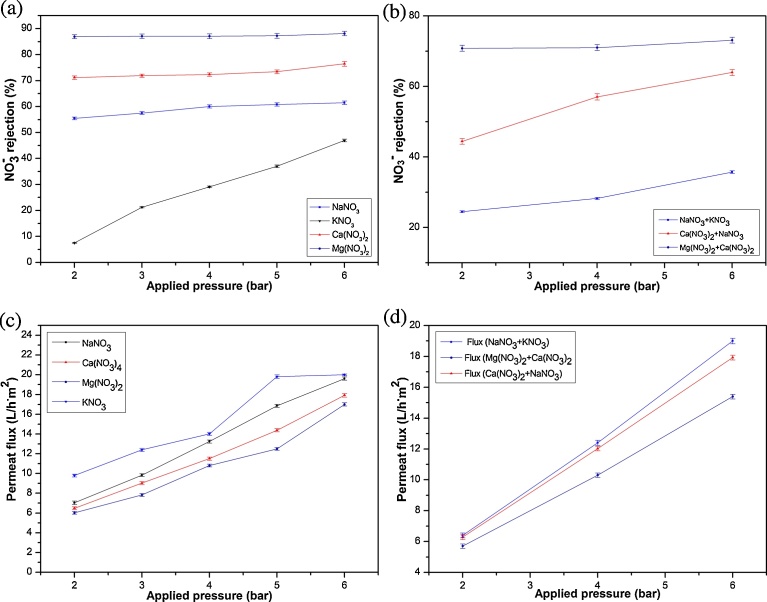
The effect of pressure on permeate flux and NO_3_^−^ retention for binary solutions KNO_3_, NaNO_3_, Ca(NO_3_)_2_, Mg(NO_3_)_2_ (a and c) and ternary solutions (NaNO_3_ + KNO_3_), (NaNO_3_ + Ca(NO_3_)_2_),(Ca (NO_3_)_2_ + Mg(NO_3_)_2_) (b and d) (C_i_ = 50 mg(NO_3_^−^)/L and natural pH).

The effect of the applied pressure on NO_3_^−^ removal and the Jp was performed at constant concentration equal to 50 mg(NO_3_^−^)/L and natural pH during 90 min of filtration (Fig. 7). The variation of applied pressure significantly affects retention behavior of NO_3_^−^ and permeate flux in binary 1:1 (NaNO_3_, KNO_3_) and 2:1 (Ca(NO_3_)_2_, Mg(NO_3_)_2_) solutions as shown in Fig. 7a and c, and in ternary solutions ((NaNO_3_ + KNO_3_), (Ca(NO_3_)_2_ + NaNO_3_) and (Ca(NO_3_)_2_ +Mg(NO_3_)_2_) as in Fig. 7b and d.

As seen in Fig. 7a and b, the retention of NO_3_^−^ from Mg(NO_3_)_2_, Ca(NO_3_)_2_ and NaNO_3_ had no significant increase with pressure (increasing from 2 to 6 bar). As known, the convection and electromigration have obvious effect on salt retention at high pressure. Whereas, the diffusion has an important role at low pressure leading to gradually decrease of KNO_3_ retention, in the reason that K^+^ have the highest diffusivity and the smallest hydrated radius (Eqs. (5) and (6)). The same behavior was observed by several authors in the case of filtration of chloride salts by *γ*-Al_2_O_3_ membrane [44, 45]. Additionally, the transfer of charged species strongly depends upon their charge and that of membrane surface. For NO_3_^−^ anion, the rejection varies according to the associated cation, and increases in the following order: K^+^ < Na^+^ < Ca^2+^ < Mg^2+^. This finding can be explained by the electrostatic repulsion of cation caused by the positive charge of the membrane at natural pH, and might be enhanced by the streaming potential generated by the act of UF. The cations are pushed toward the upstream solution while the anion is retained to keep electroneutrality in the upstream of the membrane solution. This is in agreement with the Donnan exclusion retention mechanisms [46, 47].

As shown in Fig. 7c and d, the flux also increased with increase of applied pressure.

In addition, the fluxes of single salts were weakly found to be higher than fluxes of solution with two salts. This dependency of separation on Jp was also noticed for positive charged membrane [48], neutral membrane [49] and negative charged membrane [50]. The ions transport mechanism through membranes relayed on a difference between two forces, surface force (friction and electrostatic) and convective force. When the pressure increases, surface forces stay constant while drag forces toward permeate fluxes rise. This phenomenon was caused by the increase of velocity in membrane pores. When the pressure decreases, the drag forces are less important than the surface forces. Consequently, the permeate fluxes remain low [51].

According to permeability value of membrane (5 L/m^2^ h bar), the flux values of binary solutions are lower than that of pure water which could be explained by osmotic pressure difference and by the electrostatic interaction between the membrane surface and the ions present in the solution thereby contributing to an extra resistance to the flux transfer across the membrane [30].

### 3.3 Influence of solution concentration

#### 3.3.1 Influence of solution concentration on NO_3_^−^ removal in binary solutions

**Fig. 8:**
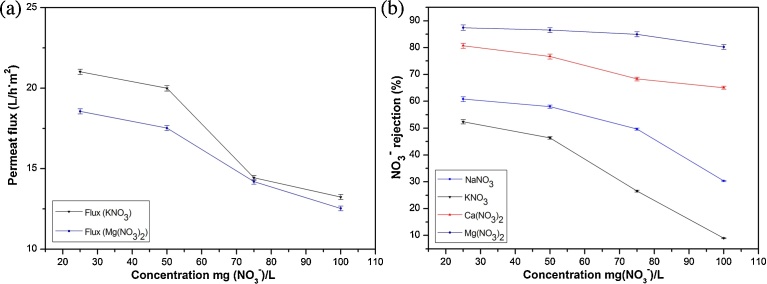
Effects of concentration on permeate flux (a) and on NO_3_^−^ rejection for binary solutions KNO_3_, NaNO_3_, Ca(NO_3_)_2_ and Mg(NO_3_)_2_ (b) (at ΔP = 6 bar, and natural pH).

Since one of the goals of UF membrane is to concentrate the effluent, it is fundamental to study the influence of the concentration on the membrane performance. Fig. 8 delineates the effect of NO_3_^−^ concentration on membrane rejection and Jp, for a fixed pressure of 6 bar and natural pH.

It is observed from Fig. 8 that higher salt concentration did not only decrease salt rejection but Jp was also depressed (Fig. 8a). The main phenomenon that could explain the decrease in flux is the osmotic pressure difference. Increase in osmotic pressure results from the increase of feed concentration. Furthermore a partial plugging of membrane at higher concentrations could happen due to formation of polarization layer [52, 53, 54].

Based on the results in Fig. 8b, the high values of NO_3_^−^ rejection were obtained for the different salts at low feed concentration of 25 mg(NO_3_^−^)/L (with nitrate rejection of 52%, 60%, 80% and 87%, respectively for KNO_3_, NaNO_3_, Ca(NO_3_)_2_ and Mg(NO_3_)_2_). This behavior is well known for charged membranes and mainly explained by the shielding phenomenon. This general attitude was explained by Kimura et al. [55], with the accentuation of the shielding effect of the effective charge of the membrane when the concentration of the counter-ions increases. Consequently, the forces of exclusion of co-ions by the surface and thus repulsive force from Donnan potential are attenuate [56, 57].

Furthermore, the electrical double layer that represents the entire interface surrounding the membrane is characterized by Debye length. An increase of the ionic strength reduces the Debye and a compression of the diffuse layer occurs, leading to a decrease of electrostatic interactions between membrane surface charge and electrolytes [34, 44].

The decrease in NO_3_^−^ rejection with an increase in salt concentration could be explained by the increasing difference in concentration between feed and permeate sides of the membrane. The presence of counter-ion near the surface of the membrane is greater than in solution, while the co-ion is more concentrated in the solution rather than surface. On account of this difference of concentration, an ion diffusion through the membrane happened [21, 56].

The weak decrease in Mg(NO_3_)_2_ rejection occurred because the charge effect stays approximately constant which could be explained either by the small effect of the membrane charge and/or by the importance of ion charge effect that does not decline at high concentration [51].

Additionally, the Jp and NO_3_^−^ rejection of different salts varied in accordance to their diffusivity and hydrated radius as shown in Table 4. The expressions given by Eqs. (5) and (6) show respectively the variation direction of Jp with cations diffusivity and the direction of NO_3_^−^ rejection with the hydrated radius of associated cations. Generally, more the ion is hydrated the more its transfer across the membrane become difficult [46, 58].

(5) $$k_{Diffusivity}^{+}>Na_{Diffusivity}^{+}>Ca_{Diffusivity}^{2+}>Mg_{Diffusivity}^{2+}$$

(6) $$k_{Hydratedradius}^{+}<Na_{Hydratederadius}^{+}<Ca_{Hydratedradius}^{2+}<Mg_{Hydratedradius}^{2+}$$

The effect of concentration on the rejection of associated cations was also measured (Table 5). Generally, higher rejections were obtained when the concentration was low for all studied cations.

**Table 5:** The rejection of associated cation in binary solutions.

Feed concentration mg (NO_3_^−^)/L	K^+^ rejection (%) in KNO_3_	Na^+^ rejection (%) in NaNO_3_	Ca^2+^ rejection (%) in Ca(NO_3_)_2_	Mg^2+^ rejection (%) in Mg(NO_3_)_2_
25	36 ± 0,358	42 ± 0,557	93 ± 0,90	97 ± 1,04
50	29 ± 0,256	39 ± 0,395	85 ± 0,846	94 ± 0,96
75	21 ± 0,242	31 ± 0,335	63 ± 0,516	92 ± 0,875
100	6 ± 0,051	20 ± 0,233	54 ± 0,433	84 ± 0,848

#### 3.3.2 Influence of feed concentration on NO_3_^−^ rejection for ternary solutions

**Fig. 9:**
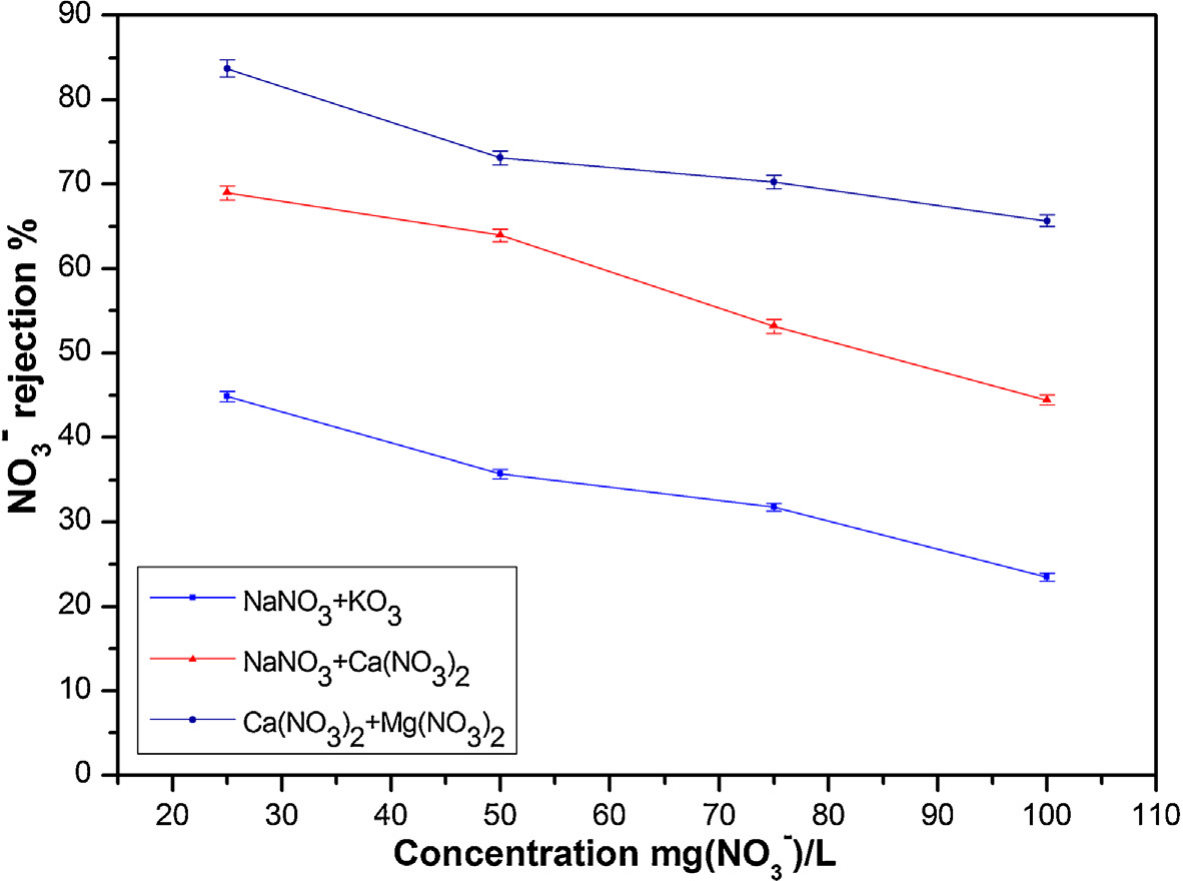
Effect of the concentration on NO_3_^−^ rejection in ternary solutions (NaNO_3_ + KNO_3_), (NaNO_3_ + Ca (NO_3_)_2_),(Ca (NO_3_)_2_ + Mg (NO_3_)_2_) (at ΔP = 6 bar, and natural pH).

The effect of initial concentration on NO_3_^−^ rejection was investigated at natural pH and pressure of 6 bar for different ternary solutions (NaNO_3_ + KNO_3_), (NaNO_3_ + Ca(NO_3_)_2_) and (Ca(NO_3_)_2_ + Mg(NO_3_)_2_) (Fig. 9).

The rejection results of ternary solutions showed lower values than binary solutions during filtration at the same concentration (regardless of the type of associated cation). This could be explained (as for binary solutions) by a decrease of the effective charge of the membrane when the concentrations increase. This decrease resulted from the dependence of the effective charge density (representing the density of the dissociated ionic groups responsible for the membrane charge) on the concentration of the salts in the feed solution.

Table 6 reports the different mixed matrix of NO_3_^−^ source using different cations and their effect on ions rejection. The NO_3_^−^ rejections were observed at high values when nitrate emanated in big amount from salts with divalent cations (Mg(NO_3_)_2_ and Ca(NO_3_)_2_) and remained below the results obtained during binary solutions filtration.

**Table 6:** Cations influence on NO_3_^−^ rejection in ternary solutions (ΔP = 6 bar and natural pH).

Ca(NO_3_)_2_ + NaNO_3_			
Ca(NO_3_)_2_ Salt %	25	50	75
NaNO_3_ Salt %	75	50	25
R(Ca^2+^)%	781 ± 0,62	75 ± 0,62	78.5 ± 0,792
R(Na^+^)%	54.9 ± 0,528	38.4 ± 0,255	38.6 ± 0,20
R(NO_3_^−^)%	65.4 ± 0,70	68.9 ± 0,580	76.5 ± 0,760

KNO_3_ + NaNO_3_			
KNO_3_ Salt %	25	50	75
NaNO_3_ Salt %	75	50	25
R(K^+^)%	5.9 ± 0,012	26.8 ± 0,2	22.5 ± 0,25
R(Na^+^)%	63.7 ± 0,570	55.1 ± 0,529	40 ± 0,425
R(NO_3_^−^)%	51.6 ± 0,462	44.8 ± 0,455	22.5 ± 0,24

Mg(NO_3_)_2_ + Ca(NO_3_)_2_			
Mg(NO_3_)_2_ Salt %	25	50	75
Ca(NO_3_)_2_ Salt %	75	50	25
R(Mg^2+^)%	89.3 ± 0,882	86.7 ± 0,862	93.3 ± 0,952
R(Ca^2+^)%	86.5 ± 0,862	84.1 ± 0,852	81.2 ± 0,792
R(NO_3_^−^)%	83.05 ± 0,819	80.6 ± 0,789	86.8 ± 0,863

The result obtained in the case of (NaNO_3_ + Ca(NO_3_)_2_) could be explained primarily by the Donnan effect [51]. The decline of Na^+^ with increasing Ca^2+^ amount is in a good agreement with charge pattern of rejection. The divalent cations were rejected more than the monovalent cations as their passage across the membrane is more difficult. This confirms that the cation valency has a dramatic effect. In addition, even though the associated cations have the same valency, the obtained rejections were dissimilar due to other factors such as hydrated radius and diffusibility that could have strong effect on NO_3_^−^ rejection.

### 3.4 UF of different mineral water doped with NO_3_^−^

Application and confirmation of previous denitrification results (during filtration of prepared solutions) was done on complex natural water using three different mineral water and by addition of 25 mg/L of NO_3_^−^. It should be noted that the mineral water samples were characterized by difference in mineralization and hardness and the added concentration of 25 mg/L of NO_3_^−^ correspond to the best NO_3_^−^ rejection for both binary and ternary solutions.

**Fig. 10:**
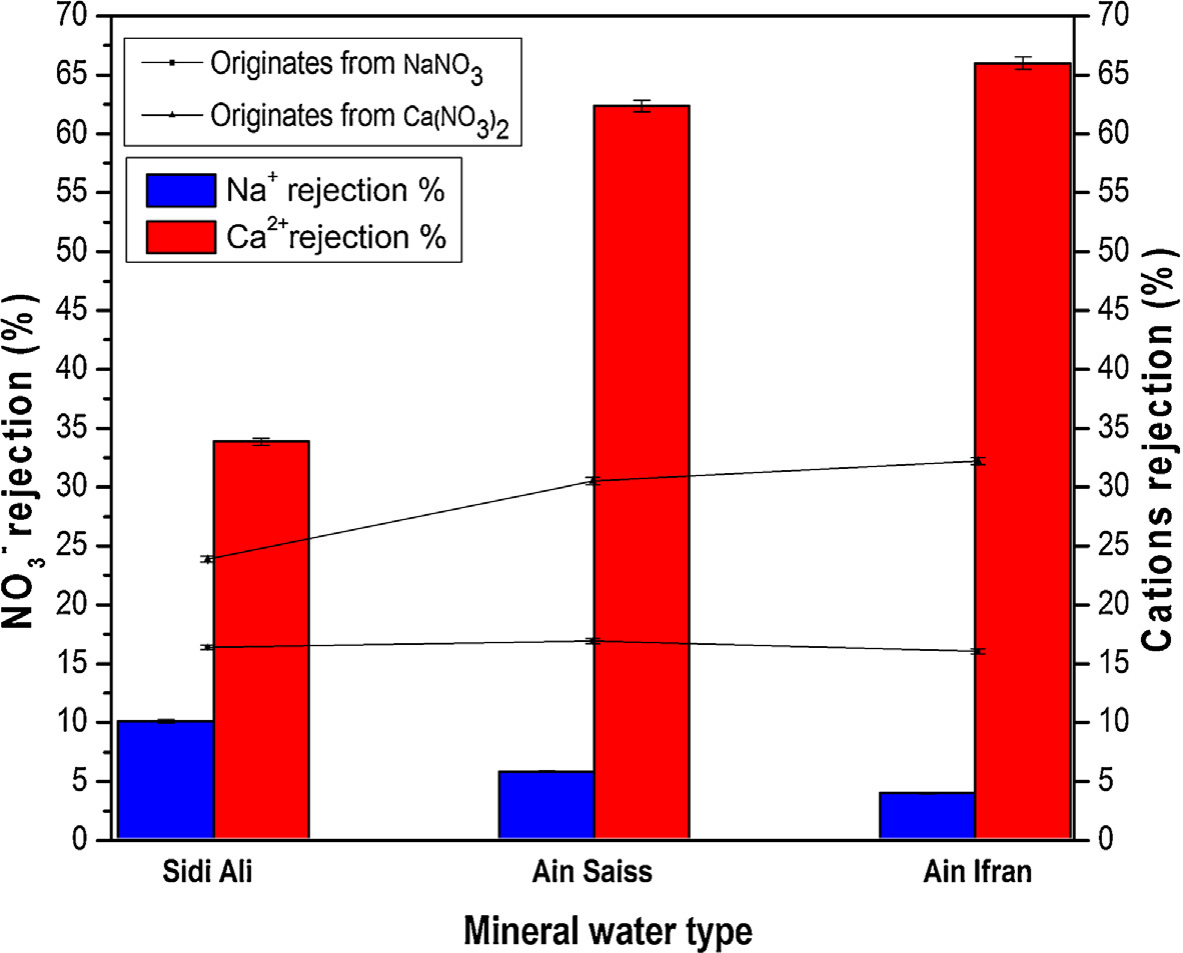
NO_3_^−^ rejection (%) as a function of the type of mineral water to be treated (C_i_ = 25 mg(NO_3_^−^)/L, ΔP = 6 bar and natural pH).

Fig. 10 displays results of filtration of different mineral water after addition of a known amount (using two nitrate salts sources) equal to 25 mg/L of NO_3_^−^, at pressure of 6 bar and natural pH. A significant difference in rejection was observed in accordance with the degree of mineralization of the used samples (Table 3). This result confirms the conclusions obtained from the study of NO_3_^−^ behavior during the filtration of prepared ternary solutions, which appears by a diminution in NO_3_^−^ rejection when the complexity of the solution is very high. In addition, the best rejection was obtained when NO_3_^−^ emanated from salt with divalent cation.

The weak rejection observed in Sidi Ali water compare to the other waters could be due to the marked presence of sulphate ions (SO_4_^2−^) (with a calculated SO_4_^2−^ rejection above 60%). In fact it has been confirmed that the SO_4_^2−^ anions are capable of reacting with AlOH_2_^+^ to form surface complexes, and the electrophoretic mobility of *γ*-Al_2_O_3_ particles decreases in contact with solution containing SO_4_^2−^. The Donnan and shielding phenomena explained once more the diminution of nitrate rejection [37, 59].

## 4 Conclusion

This study focused on the use of *γ*-Al_2_O_3_ UF membrane in nitrate removal from prepared solutions and commercial mineral water. The experimental parameters such as pH, applied pressure, initial concentration of NO_3_^−^ and cation valency were investigated in order to evaluate the membrane performance.

The NO_3_^−^ rejection was optimal when adding divalent cations rather than monovalent cations due to a big hydration radius causing important repulsion.

The selectivity of the membrane strictly depended upon the feed pH which changed the membrane charge (amphoteric character: positive in acid medium and negative in basic medium). The high rejection of NO_3_^−^ was obtained around pH_pzc_. Moreover, the presence of some ions (Ca^2+^) may have shifted the pzc of the membrane and thus varying the membrane performance.

The experimental results showed that an increase of NO_3_^−^ concentration in the feed solution declines the NO_3_^−^ rejection caused by a membrane shielding phenomenon which reduces the interaction between the ionic solute and the membrane. Meanwhile, the effect of applied pressure on NO_3_^−^’s removal indicated that the rejection increased with pressure due to osmotic pressure difference.

The efficiency of NO_3_^−^ filtration strictly depended upon the complexity of the ionic composition of the solution, and the influence of parameters such as the solute hydration radius and its energy, and the interaction solute-solute and membrane-solute.

The filtration of three commercial mineral water samples containing added NO_3_^−^ (50 mg/L of NO_3_^−^), revealed that increasing the complexity of water decreased the rejection of NO_3_^−^ thereby conforming the results obtained for synthetized ternary solutions. In addition, the best NO_3_^−^ rejection is found for water presenting a low total mineralization and a low SO_4_^2−^ ions concentration.

Finally, the *γ*-Al_2_O_3_ UF membrane could be used as alternative treatment process for denitrification of contaminated water especially in agricultural areas due to the intensive use of fertilizer.

## Declarations

### Author contribution statement

M. Breida: Performed the experiments; Analyzed and interpreted the data; Wrote the paper.

S. Alami Younssi: Conceived and designed the experiments.

A. Bouazizi: Performed the experiments.

B. Achiou: Analyzed and interpreted the data.

M. Ouammou and M. El Rhazi: Contributed reagents, materials, analysis tools or data.

### Competing interest statement

The authors declare no conflict of interest.

### Funding statement

This work was supported by MESRSFC (Ministère de l'Enseignement Supérieur et de la Recherche Scientifique et de la Formation des cadres – Morocco) and CNRST (Centre National pour la Recherche Scientifique et Technique – Morocco) (Project number PPR/2015/72).

### Additional information

No additional information is available for this paper.
